# Med-Diffusion: Diffusion Model-Based Imputation of Multimodal Sensor Data for Surgical Patients

**DOI:** 10.3390/s25196175

**Published:** 2025-10-05

**Authors:** Zhenyu Cheng, Boyuan Zhang, Yanbo Hu, Yue Du, Tianyong Liu, Zhenxi Zhang, Chang Lu, Shoujun Zhou, Zhuoxu Cui

**Affiliations:** 1 Shenzhen Institute of Advanced Technology, Chinese Academy of Sciences, Shenzhen 518055, China; zy.cheng@siat.ac.cn (Z.C.); 17852156693@163.com (B.Z.); yb.hu2@siat.ac.cn (Y.H.); yue.du2@siat.ac.cn (Y.D.); ty.liu1@siat.ac.cn (T.L.); zx.zhang3@siat.ac.cn (Z.Z.); 2 University of Chinese Academy of Sciences, Beijing 100049, China; 3 Department of Mathematics, University of Shanghai for Science and Technology, 334 Campus, Shanghai 200093, China; 254132139@st.usst.edu.cn

**Keywords:** sensor data enhancement, medical information, diffusion models, deep learning

## Abstract

The completeness and integrity of multimodal medical data are critical determinants of surgical success and postoperative recovery. However, because of issues such as poor sensor contact, small vibrations, and device discrepancies during signal acquisition, there are frequent missing values in patients’ medical data. This issue is especially prominent in rare or complex cases, where the inherent complexity and sparsity of multimodal data limit dataset diversity and degrade predictive model performance. As a result, clinicians’ understanding of patient conditions is restricted, and the development of robust algorithms to predict preoperative, intraoperative, and postoperative disease progression is hindered. To address these challenges, we propose Med-Diffusion, a diffusion-based generative framework designed to enhance sensor data by imputing missing multimodal clinical data, including both categorical and numerical variables. The framework integrates one-hot encoding, simulated bit encoding, and feature tokenization to improve adaptability to heterogeneous data types, utilizing conditional diffusion modeling for accurate data completion. Med-Diffusion effectively learns the underlying distributions of multimodal datasets, synthesizing plausible data for incomplete records, and it mitigates the data sparsity caused by poor sensor contact, vibrations, and device discrepancies. Extensive experiments demonstrate that Med-Diffusion accurately reconstructs missing multimodal clinical information and significantly enhances the performance of downstream predictive models.

## 1. Introduction

Multimodal data from surgical patients, encompassing preoperative, intraoperative, and postoperative information, play a crucial role in ensuring successful surgeries and improving patient prognosis [1,2]. These data offer invaluable insights into patients’ physiological status, potential risk factors, and responses to various surgical and anesthetic interventions and treatments. The effective utilization of cardiovascular data can inform clinical decision making, guide personalized treatment strategies, and optimize resource allocation in healthcare settings [3].

However, the acquisition of comprehensive cardiovascular data for patient risk assessment often encounters numerous challenges. Due to technical limitations, disease progression, and other factors, some data modalities may be missing or uncollected, which affects the accuracy and reliability of the assessment [4,5,6]. Complete and accurate modality data is vital for constructing effective predictive models, as missing data can lead to the loss of critical information or introduce bias during the risk evaluation process. Healthcare data imputation not only improves the utilization of existing data—maximizing available resources—but also enhances the efficiency of model training [7]. Risk assessment of patients during surgery usually requires the integration of multiple modalities to gain a comprehensive understanding of a patient’s health condition. Missing modality data can limit the model’s ability to fully analyze the patient’s status [8]. There are imputation techniques that generate missing modality data and align them with existing data ensure data integrity, thereby improving the reliability and accuracy of predictive models.

By exploring methods for generating and aligning missing data, we can effectively impute the missing data and integrate it with other modalities [9], facilitating the synergistic use of multimodal data and enhancing the comprehensiveness and accuracy of the evaluation.

Data augmentation is a powerful technique to address the issue of data scarcity. Researchers have explored the potential of medical data augmentation to compensate for the limited datasets of surgical patients [10]. Recent advancements in generative models have shown promising results in generating high-quality synthetic data, such as MissForest Random Forest Imputation [11] and GAINs (Generative Adversarial Imputation Networks) [12], which can significantly augment existing datasets [13]. Specifically, diffusion models have been proven to outperform Generative Adversarial Networks (GANs) in image generation tasks, offering advantages, such as broader distribution coverage, stable training objectives, and strong scalability [14,15]. The application of generative models in medical data augmentation has increasingly drawn the attention of researchers [16]. By leveraging these technologies, healthcare providers can overcome missing data issues and build more accurate and reliable predictive models [17], supporting clinical decision making and improving the care of surgical patients [18]. Integrating diffusion-based augmentation techniques into multimodal medical data workflows enhances data availability and diversity, thereby enabling informed clinical decisions, personalized treatments, and improved patient outcomes [19].

To address the issue of missing data in multimodal medical datasets, we propose Med-Diffusion, a diffusion model-based conditional imputation and data augmentation framework designed to tackle missing and sparse data challenges in multimodal medical data. This method is carefully designed to consider both the complexity of multimodal features and the practical challenges of data scarcity and frequent missing data in medical settings. First, the framework preprocesses tabular data and constructs training missing data based on a Missing Completely at Random (MCAR) mechanism to ensure that the model learns more robust imputation strategies in a controlled environment. At the representation layer, the framework systematically compares three different approaches for handling categorical variables: one-hot encoding, simulated bit encoding, and feature tokenization (FT, feature embedding). The FT approach, which demonstrated superior performance, was ultimately selected. This approach maps both numerical and categorical variables into the same-dimensional embedding space, allowing the entire multimodal data to be fully numerically represented and input into the diffusion model. Subsequently, Med-Diffusion performs a reversible “noising → denoising” process: during the training phase, the model learns a conditional denoising network on complete data using the observed sub-vectors as conditions and minimizing the mean squared error (MSE) of the noise prediction to approximate the true distribution. During the inference phase, the model only performs stepwise denoising on the missing dimensions of incomplete samples, while keeping the observed dimensions unchanged. The imputation result is then obtained through the decoding strategy corresponding to one-hot encoding, simulated bit encoding, or FT. This process allows the framework to handle both numerical variables (such as heart rate, systolic/diastolic blood pressure, temperature, and respiratory rate) and categorical variables (such as cancer stages I–IV and smoking status at light/moderate/severe), ensuring the imputation’s plausibility while enhancing data diversity and utilization. Compared to traditional methods, Med-Diffusion not only provides a more comprehensive and flexible solution, but also demonstrates clear advantages in terms of training stability, distribution coverage, and the quality of generated samples. Experimental results, assessed using RMSE and error rates, validate the framework’s effectiveness and its competitiveness relative to state-of-the-art methods.

**Figure 1:**
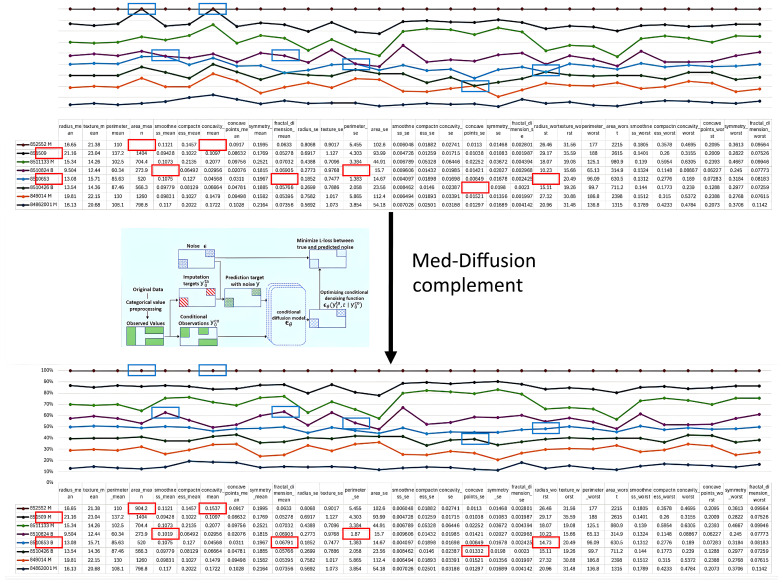
To address the issue of missing data arising from the heterogeneity of sensors and medical devices, we propose Med-Diffusion. The red box indicates the comparison table of data before and after imputation, and the blue box represents the comparison line charts of data imputation across different examples. The imputation process and the corresponding results obtained by Med-Diffusion are illustrated in Figure 1. Med-Diffusion effectively learns the underlying distributions of multimodal datasets and synthesizes plausible data for incomplete records, thus improving overall data quality and utility.

Our primary contributions are as follows. The experimental results are shown in Figure 1.

- We propose a novel diffusion model-based multimodal medical data imputation method, Med-Diffusion, and implement it for the tabular imputation of static medical sequences.
- We propose a comprehensive pipeline for effectively training Med-Diffusion on categorical features, utilizing advanced encoding and embedding techniques.
- Our experimental results show that Med-Diffusion reduces error rates and the RMSE (Root Mean Square Error) by 4.4% and 5.1%, respectively, compared to existing probabilistic methods for healthcare and environmental data.

## 2. Related Works

### 2.1. Disease Risk Prediction Based on Multimodal Medical Data

In recent years, with the rapid development of machine learning and deep learning technologies, disease risk prediction based on multimodal medical data has become an important research direction in the medical field. Many studies have combined preoperative, intraoperative, and postoperative patient data with machine learning models to improve the accuracy of disease risk prediction. These studies used various deep learning models to learn task-relevant information from high-dimensional features, particularly achieving significant progress in the fusion and analysis of multimodal data.

For example, Chen et al. [20] proposed a deep learning model based on Long Short-Term Memory (LSTM) networks to predict postoperative complication risks based on preoperative physiological indicators and laboratory test results. This approach effectively captures the long-term dependencies in time series data, but its limitation lies in the sensitivity of the LSTM model to the sequence of input data and its difficulty in handling the complex relationships between different modalities. In contrast, Hui et al. [21] developed an ensemble model that combines various machine learning algorithms to predict the risk of bleeding after cardiac surgery. The advantage of this method is its ability to integrate the strengths of multiple algorithms, but the model’s robustness still needs improvement when dealing with incomplete data or highly heterogeneous data. Weng et al. [22,23] employed neural networks to perform intelligent analysis of patient imaging data to assess surgical risk. Although this method performed excellently on imaging data, it showed poor adaptability to non-imaging data, such as physiological indicators and laboratory test results.

In addition, recent studies have begun to explore the application of Graph Neural Networks (GNNs) in disease risk prediction. For instance, Rao et al. [24] proposed a GNN-based model that combines Electronic Health Records (EHRs) and medical imaging data to predict postoperative complications in surgical patients. This method effectively modeled the complex dependencies between multimodal data, but its computational complexity is relatively high, and there may be efficiency bottlenecks when dealing with large-scale datasets. Meanwhile, the introduction of transfer learning and domain adaptation techniques has effectively improved the model’s generalization ability in different medical scenarios. For example, through transfer learning, pre-trained models can be effectively adapted to specific cardiovascular and cerebrovascular datasets, thereby improving the accuracy of disease risk prediction.

Overall, although the above studies have made certain progress in multimodal data fusion and disease prediction, most methods still face challenges, such as data heterogeneity, missing data, and model computational complexity. Future research should focus more on how to efficiently handle multi-source heterogeneous data and improve the model’s generalization ability in real-world applications.

### 2.2. Multimodal Medical Data Imputation

Multimodal medical data imputation is a key step in disease risk prediction, as multimodal medical datasets often contain large amounts of missing data. Traditional imputation methods, such as mean or median imputation and multiple imputation based on statistical learning, can fill in some missing data, but they ignore any correlations between features, assuming that each feature is independent. This leads to significant bias in the imputed data distribution, distorting the true relationships between variables. As a result, when dealing with high-dimensional and complex medical data, these methods often underperform, leading to a significant decline in the performance of the subsequent analysis models (e.g., disease prediction models) based on this data. In recent years, generative artificial intelligence models, such as Variational Autoencoders (VAEs) and MissForest (Random Forest Imputation), have been widely used in medical data imputation due to their powerful data generation capabilities.

For example, MissForest, as a non-parametric imputation method, has gained widespread use in recent years. MissForest handles missing values through a random forest model. It is an iterative imputation method that uses the conditional distribution of known data to predict missing values. Unlike traditional imputation methods (such as mean imputation and MICE), MissForest does not rely on any specific distribution assumptions. Instead, it trains a random forest to learn the conditional distribution of each feature, updating the imputation results in each iteration based on the current predicted missing values. The main distinction of MissForest from other imputation methods lies in its flexibility and non-parametric nature. Compared to traditional imputation methods based on statistical assumptions, MissForest can automatically adapt to the data’s structure and better capture non-linear relationships between variables. For example, traditional MICE methods use linear regression models to impute missing values, but its iterative process may struggle to converge generally or converge to suboptimal solutions when encountering highly collinear features or poorly fitting prediction models for some features. MissForest, on the other hand, processes the complex relationships in the data through random forests, exhibiting stronger robustness. However, although MissForest performs well in many practical problems, it is an iterative process. Despite providing good imputation results, each iteration requires training a complete random forest for each missing feature, leading to high computational costs, particularly when dealing with high-dimensional, large-sample medical data (making scalability an issue). Furthermore, MissForest’s performance is highly dependent on the feature relationships in the data. If there are extreme missing patterns or high heterogeneity in the data, the imputation accuracy of MissForest may be affected.

Meanwhile, Ilias et al. [25] proposed a VAE-based imputation method for filling in missing values in Electronic Health Records (EHRs). This method generates reasonable imputed values by learning the latent space distribution of the data, but its assumptions about the data distribution may lead to biased imputation results. Peng et al. [26] introduced a framework based on Recurrent GANs (RecurrentGANs), which learns data distributions from time series data and imputes missing continuous waveform signals, demonstrating its potential in handling time series data. However, GAN-based models, such as GAINs, are known for their instability during training and difficulty in converging, often falling into mode collapse, where the generator produces only a few seemingly reasonable imputed values with insufficient diversity. In contrast, Ahn et al. [27] explored the use of diffusion probabilistic models to generate synthetic data, enhancing patient outcome prediction performance. Although this method can effectively generate high-quality synthetic data, it involves significant computational overhead and may encounter issues, such as prolonged training times in real-world applications. Recent studies have introduced attention mechanisms and Transformer models into multimodal data imputation. Xie et al. [28] proposed an attention-based VAE model that adaptively weights different variables to impute missing values in EHRs. Although this method improved imputation accuracy, the latent space distribution modeling assumption of the VAE model may not be suitable for handling complex dependencies or special missing patterns in the data, leading to inaccurate imputation results. Additionally, the model’s training process is complex, with issues such as vanishing gradients, overfitting, and instability during training with high-dimensional data. Furthermore, since the imputed values generated by VAE are sampled from the latent space, the results may deviate from the true data distribution, especially when dealing with complex missing patterns.

Therefore, future research needs to explore how to improve computational efficiency while ensuring high-quality imputation and how to handle larger-scale medical data.

### 2.3. Deep Learning-Based Data Imputation

In recent years, deep learning methods have demonstrated superior performance over traditional statistical methods in time series data imputation, particularly in modeling the temporal dependencies of data. Recurrent Neural Networks (RNNs) and their variants, such as Long Short-Term Memory networks (LSTMs) and Gated Recurrent Units (GRUs), have been widely used for data modeling [18]. Compared to traditional linear imputation methods, RNNs are better at capturing the sequential features of data, leading to better performance in imputation tasks, such as GANs and self-training strategies [29].

The GAINs method [14] is a deep generative model based on GANs, where the missing data imputation task is treated as an adversarial learning problem. The GAINs framework includes a generator and a discriminator: the generator receives input with masks (missing values are randomly set as noise) and attempts to generate reasonable imputed values, while the discriminator learns to distinguish real data from generated data. Through this adversarial process, GAINs is able to learn more complex joint distributions and higher-order correlations, making it advantageous for high-dimensional heterogeneous data. However, as a variant of GANs, GAINs’ training process requires substantial computational resources and may face the issue of mode collapse, especially in high-dimensional data imputation tasks.

Among these methods, models incorporating attention mechanisms stand out in handling imputation tasks [30]. However, most of these methods are deterministic imputation methods and fail to fully account for the uncertainty present during the imputation process. It was not until the introduction of GP-VAE [30] that uncertainty modeling was incorporated into imputation tasks, allowing the imputed results to better reflect the underlying diversity of the data distribution. Despite this, current deep learning-based imputation methods still face two major challenges: first, how to better integrate the complex characteristics of time series data with other types of data (such as categorical data); second, how to address computational efficiency issues when handling large-scale datasets. Future research should explore the integration of deep learning with other imputation methods to improve imputation accuracy while reducing computational overhead.

### 2.4. Generative Artificial Intelligence and Diffusion Methods

Generative artificial intelligence has made significant progress in various fields in recent years, especially in image generation [31,32,33], audio synthesis [34,35], and graph-structured data generation [36]. By introducing score-matching strategies based on Langevin dynamics or Denoising Diffusion Probabilistic Models (DDPMs), these methods can effectively learn the data distribution and generate high-quality samples.

In the time series domain, TimeGrad [37,38] employs a diffusion model for probabilistic forecasting, achieving state-of-the-art performance. However, due to its reliance on Recurrent Neural Networks (RNNs) to process past temporal information, it has certain limitations when handling missing value imputation tasks. Despite this, the advantages of diffusion models in fields such as image generation provide new insights, particularly in medical data imputation tasks. By mapping data to latent space and modeling the joint distribution, diffusion models can more flexibly capture complex relationships between variables, offering a novel solution for missing data imputation in medical datasets. While generative artificial intelligence methods have achieved remarkable results in several fields, how to fully leverage the advantages of diffusion models and integrate them with traditional imputation methods for medical data imputation remains an area worthy of in-depth research.

## 3. Methodology

We propose a multimodal medical data augmentation method based on diffusion models. The core idea of this approach is to treat the process of data generation as a gradual transition from data distribution to a Gaussian distribution. This process can be conceptualized as the data progressively losing its structural characteristics and eventually becoming disordered, analogous to the diffusion phenomenon in physics. During the generation of new samples, the model must reverse this process, gradually restoring the ordered data structure from the disordered Gaussian distribution. Experimental results are shown in Table 1.

### 3.1. Overview

For multimodal medical data imputation, the proposed method includes four main stages, executed as follows.

1. Handling Categorical Variables: Categorical variables are processed using one-hot encoding, simulated bit encoding, and embedding encoding methods. Among these, the embedding method (feature tokenization/FT) is found to perform better. Thus, categorical variables are processed using FT in the experimental implementation, and this is reflected in Table 2.
2. Forward Diffusion Process: Both the complete dataset and the incomplete dataset are gradually transformed into a Gaussian distribution by adding noise, which generates a series of progressively noisy states.
3. Reverse Generation Process: A neural network is trained on the complete dataset to model the reverse process. This process enables the model to gradually recover the original data from noisy states, essentially reversing the effects of the forward diffusion process.
4. Imputing Missing Data: For the incomplete dataset, the reverse process, which is trained on the complete dataset, is used to gradually recover the missing data points from the Gaussian noise state. This process combines the reverse diffusion model with the conditional observed values.

Before handling categorical variables, data preprocessing is required. Preprocessing mainly involves distinguishing between categorical and numerical variables, as well as randomly masking some feature entries to construct training and evaluation samples. Specifically, this study employed the Missing Completely at Random (MCAR) mechanism to mask the original data. The mechanism operates as follows: for each row of data, a random subset of the originally observable entries is uniformly selected and masked. Specifically, for each row of data, we randomly select $\left\lfloor N_{\mathrm{obs}}\left(\mathrm{col}\right)\times 0.2\right\rfloor$ entries from the non-missing samples to mask (rounded down). Therefore, unless the dataset is particularly small, the actual missing rate for all features is roughly the same. If a row of data has no missing entries initially, the final missing rate for that row is 20%. If some entries are already missing, then the final missing rate for that row is slightly above 20%.

To facilitate subsequent training and evaluation, we placed all continuous features (numerical variables) at the beginning of the data, while categorical features (categorical variables) were placed at the end. This arrangement does not affect the masking mechanism, as all columns are independently masked in a uniformly random manner. Additionally, the randomness of the entire process is controlled by a fixed random seed to ensure reproducibility of the results.

**Figure 2:**
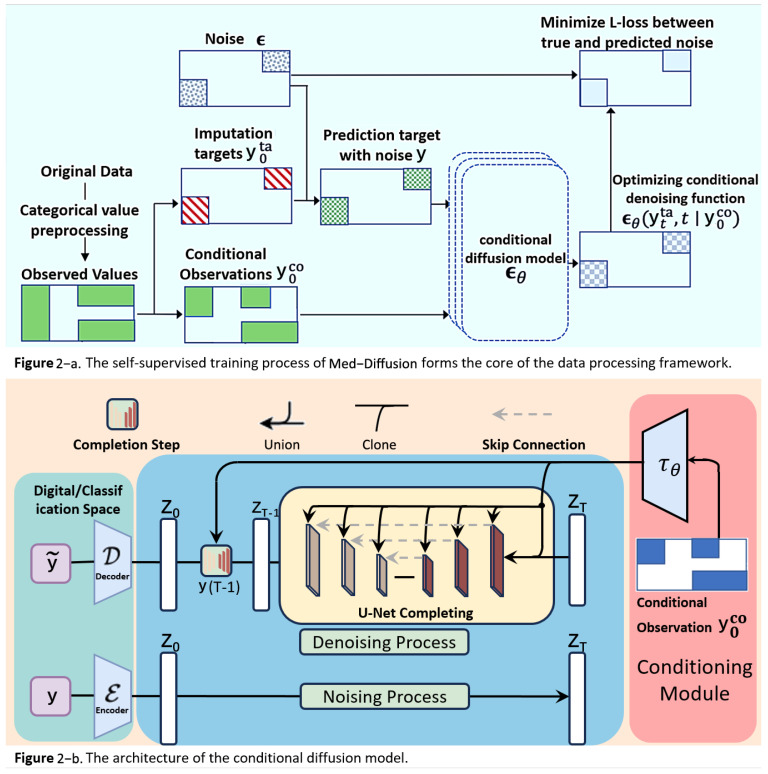
(**a**) In the rectangle on the middle left, the green and white areas represent observed and missing values, respectively. The observed values are divided into the red imputation target $y_{0}^{\mathrm{ta}}$ and the blue conditional observed values $y_{0}^{\mathrm{co}}$ , and they are used to train $\epsilon_{\theta }$ . The colored regions in each rectangle represent the presence of values. (**b**) During the training process, the training dataset randomly masks some data as the prediction target data, and multiple training epochs are used to compute and minimize this loss. During inference, the model with the minimum loss is used for predictions, with the actual missing data serving as the prediction target data.

Moreover, the process treats each row of data the same way, applying the same missing rate to all features of each row, meaning that the missing rate for each feature is approximately the same. To make processing more convenient for subsequent training and evaluation, we place continuous variables (numeric features) in the front and categorical variables (categorical features) in the back. This does not affect the handling of missing values because all data columns independently follow the same MCAR missing pattern. The framework of the Med-diffusion and the training process are shown in Figure 2.

### 3.2. Categorical Variable Processing

Before processing categorical variables, it is necessary to distinguish between categorical and numerical variables. During the preprocessing stage, we explicitly specify which columns are categorical variables for encoding, while the remaining data is treated as numerical variables, skipping the processing of categorical variables for missing data imputation.

**Figure 3:**
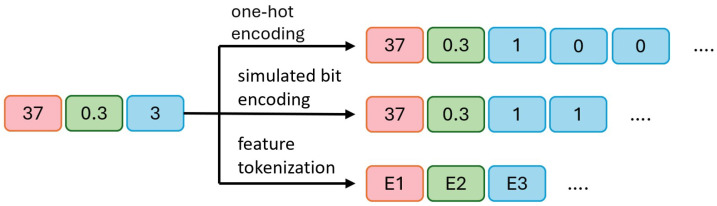
An example of handling categorical variables using one-hot encoding, simulated bit encoding, and feature tokenization. The two numerical variables in the example are labeled in red and green, while the categorical variable is labeled in blue. It is good practice to explain the significance of the figure in the caption.

To effectively handle categorical variables, this method explores three techniques (as shown in Figure 3): one-hot encoding, simulated bit encoding, and feature tokenization. In the original data, 45 and 0.7 are numerical variables (represented by blue and green blocks), while 3 is a categorical variable (represented by a yellow block), assuming there are three possible categories.

a. In one-hot encoding, 45 and 0.7 remain unchanged, while the categorical variable 3 is converted into three binary bits, [1, 0, 0], indicating the third category is selected (1) and the other two are unselected (0).
b. In simulated bit encoding, 45 and 0.7 remain unchanged, with each category assigned a unique fixed-length binary code. The categorical variable 3 is encoded as two binary bits, [1, 1], where a more compact representation requires fewer bits.
c. In processing categorical variables, embedding encoding is used to convert all variables (both numerical and categorical) into embeddings of the same length. E1, E2, and E3 represent the embedded representations of the three original variables. This approach maps all variables to a unified dimensional space. During output processing, the embedding vector is converted back to the original numerical or categorical values. For numerical variables, the final value is obtained by averaging the elements of the embedding vector divided by the output of the diffusion model. For categorical variables, the predicted category is determined by calculating the distance between the embedding vector and the diffusion model output, selecting the nearest embedding vector.

### 3.3. Diffusion Probabilistic Model for Numerical Imputation

The framework of this study is primarily based on diffusion models, which serve as the mathematical foundation for both categorical and numerical imputation tasks, enabling a seamless transition between noisy and clean data. The following diffusion probabilistic model serves as the mathematical foundation for numerical imputation in this paper. It defines how data progressively transitions from an ordered state to a disordered state, and it also describes the process for generating data in reverse. The key components of the diffusion probabilistic model are as follows (the detailed schematic illustration is provided in Figure 2).

#### 3.3.1. Objective and Evaluation Metric

Let $Y={\left(R\cup \left\{\emptyset \right\}\right)}^{d}$ represent the input space, where R denotes the real number space and *∅* represents missing values. In the missing value imputation task, we are given a *d*-dimensional training dataset $Y_{tr}={\left\{y_{i}\right\}}_{i=1}^{n}$ , where *n* is the number of data points. Without loss of generality, the *j*-th feature of data point $y_{i}$ (with j∈{1,…,d} ) is defined as $y_{i}^{j}\in Y$ , and this feature can be a missing value, numerical variable, or categorical variable. This study focused on inductive learning (a type of learning where general rules or models are derived from training data and applied to unseen data) with the goal of finding an imputation function $f:Y\to R^{d}$ , which will allow the conversion of the input space *Y* with missing values into a *d*-dimensional real-valued space, replacing missing values with reasonable values. The specific steps for finding the imputation function include the following.

(1) **Defining the Conditional Diffusion Model:** Med-Diffusion introduces the Conditional Diffusion Model to impute the missing values conditioned on observed data. This includes the following. Conditioning Setup: For each sample $y_{i}$ , define the observed part $y_{0}^{co}$ (non-missing values) and the missing part *y*. The goal is to learn the conditional distribution $p(y|y_{0}^{co})$ . In the diffusion model, the conditional information $y_{0}^{co}$ is incorporated into the reverse process. Forward Process: The forward process is the same as in the standard diffusion model, where noise is gradually added to the true data $y_{0}$ , generating $Y_{1},\ldots ,Y_{T}$ . For numerical variables, Gaussian noise is directly added; for categorical variables, one-hot encoding is applied first, and then noise is added. Reverse Process: The reverse process is conditioned on the observed values $y_{0}^{co}$ and progressively denoised to generate imputed values. Med-Diffusion modifies the reverse process to focus on imputing the missing part while keeping the observed part unchanged.
(2) **Processing Numerical and Categorical Variables:** Med-Diffusion employs distinct modeling strategies for numerical and categorical variables:
  a. Numerical Variables (e.g., heart rate and blood pressure). Modeling: Numerical variables are treated as continuous values, fitting the Gaussian noise assumption of the diffusion model. Imputation: During the reverse diffusion process, missing numerical values are progressively denoised to reasonable real values. For example, heart rate might recover from a noisy value to 80 beats per minute, and blood pressure may return to 120/80 mmHg. Loss Function: Mean squared error (MSE) is used to optimize the imputation results, ensuring the imputed values are close to the true distribution.
  b. Categorical Variables (e.g., alcohol consumption level and heart rhythm status). Modeling: Categorical variables are transformed into continuous vectors through one-hot encoding. However, the Gaussian noise of the diffusion model may disrupt the discrete nature. Therefore, Med-Diffusion introduces a Discrete Diffusion Model or a post-processing step. Imputation: During the reverse diffusion process, the one-hot (embedding) vector for categorical variables is denoised, generating a probability distribution (e.g., alcohol consumption level might produce [0.7, 0.2, 0.1]). Post-processing: The probability distribution is converted into discrete categories by selecting the category with the highest probability (e.g., [0.7, 0.2, 0.1] becomes “mild”). Loss Function: Cross-entropy loss is used to optimize the imputation results for categorical variables, ensuring the imputed category aligns with the true distribution.
(3) **Training the Imputation Function *f*:** Neural Network Design: Med-Diffusion employs a neural network based on a diffusion architecture to parameterize the mean $\mu_{\theta }\left(y_{t},t\right)$ of the reverse diffusion process. Input: Current time step *t*, noisy data $y_{t}$ , and conditional information $y_{0}^{co}$ . Output: Denoised data $y_{t-1}$ , which corresponds to the imputed data. Training Goal: The goal is to optimize the neural network parameters θ so that the predicted noise approaches the true noise, thus making the denoising process as realistic as possible. The objective function is
(1) $$f_{\mathrm{sample}}\left(\theta \right)=E_{t,y_{0},\epsilon }\left[∥\epsilon -\epsilon_{\theta }\left(y_{t},y_{0}^{co}\right){∥}^{2}\right],$$
  where $\epsilon_{\theta }$ is the noise predicted by the neural network, and $y_{0}^{co}$ represents the conditional information. t∼Uniform{1,…,T} : A time step *t* is randomly selected from the diffusion process. $y_{0}∼q\left(y_{0}\right)$ : An original data point $y_{0}$ is sampled from the training set, which may contain missing or non-missing values. ϵ∼N(0,I) : Noise sampled from a standard normal distribution. $y_{t}=\sqrt{\alpha_{t}}y_{0}+\sqrt{1-\alpha_{t}}\epsilon$ : Given an original sample $y_{0}$ and a time step *t*, the noisy version $y_{t}$ is obtained using this formula. $\alpha_{t}$ is a time-dependent noise decay factor, which is predetermined in this experiment (usually provided by a pre-defined linear noise schedule). $\epsilon_{\theta }\left(y_{t},y_{0}^{co}\right)$ : The model predicts the most likely original noise ϵ given the noisy data $y_{t}$ , the current time step *t*, and the conditional information $y_{0}^{co}$ (i.e., the observed non-missing part). $∥\epsilon -\epsilon_{\theta }\left(y_{t},y_{0}^{co}\right){∥}^{2}$ : The standard mean squared error (MSE) between the model’s predicted noise and the true noise. Minimizing this error is equivalent to instructing the model to “reverse the noise I added.” **Training Process.** a. Sample $y_{0}$ from the training data $Y_{tr}$ . b. Randomly select a time step *t* and generate the noisy version $y_{t}$ . c. Use the observed values $y_{0}^{co}$ as conditioning information to predict the noise $\epsilon_{\theta }$ . d. Optimize the loss function and update the network parameters θ .
(4) **Inference Process (Generation Process):**
  a. **Input Sample.** The input sample $y_{\mathrm{new}}$ , which contains missing values, is represented as $y_{\mathrm{new}}\in {\left(R\cup \left\{\emptyset \right\}\right)}^{d}$ . This vector has several missing dimensions to be predicted (denoted by a mask *M*, where $M_{j}=0$ indicates the *j*-th dimension is missing and $M_{j}=1$ indicates the *j*-th dimension is observed). We divided the data into two parts—the conditional observed values $y_{0}^{co}$ as the observable part and the target missing values *y* as the part to be imputed. The observed and missing sections are represented by the blue and red areas in Figure 2.
  b. **Initialization of Missing Value Region, Forming $z_{T}$ .** To initiate the reverse diffusion process, we need an initial “fully noisy” vector. The observed region retains the original $y_{0}^{co}$ ; the missing region *y* is initialized with random noise (Gaussian distribution). We constructed an initial state $z_{T}$
(2) $$z_{T}=\sqrt{{\bar{\alpha }}_{T}}y_{0}+\sqrt{1-{\bar{\alpha }}_{T}}\epsilon ,$$
    where ϵ∼N(0,I) , introducing noise in only the missing region, while the observed region retained the true values.
  c. **Conditional Control Mechanism Input—Feeding into Multi-layer Denoising U-Net for** *T* **Steps.** Next, we feed the current noisy data $z_{t}$ , the current time step *t*, and the conditional observed values $y_{0}^{co}$ into a neural network function $\epsilon_{\theta }$ (in this paper, U-Net). θ represents the network parameters, and the network predicts the noise term $\epsilon_{\theta }$ . In other words, the network asks the following: “How should I remove the noise to make the missing region more realistic?”
(3) $$\epsilon_{\theta }=\epsilon_{\theta }\left(z_{t},t,y_{0}^{co}\right)$$
    The “conditioning mechanism” (highlighted in red in the figure) is crucial as it ensures that the network knows which areas are the observed values and which are missing, allowing for the imputation of the missing values according to the completion process of the U-Net.
  d. **Step-by-Step Reverse Diffusion, from $z_{T}$ to $z_{0}$ .** Using the noise output $\epsilon_{\theta }$ from the forward process, we update according to the reverse formula
(4) $$z_{t-1}=M⊙y_{0}+\left(1-M\right)⊙\left(\frac{1}{\sqrt{\alpha_{t}}}\left[z_{t}-\frac{1-\alpha_{t}}{\sqrt{1-{\bar{\alpha }}_{t}}}\epsilon_{\theta }\left(z_{t},t,y_{0}^{co}\right)\right]+\sigma_{t}\epsilon \right)\;\epsilon ∼N\left(0,I\right),$$
    Note that the observed region is “condition-locked” and does not participate in sampling or noise updating. The reverse diffusion mechanism generates the predicted missing values based on the conditional observed data. Starting from t=T , we update step-by-step to t=0 , gradually denoising the data from a fully noisy state to the true data space, generating the imputed values for the missing regions.
  e. **Obtaining the Imputed Result $\tilde{y}$ .** Finally, when t=0 , the network outputs the predicted values for the features $z_{0}$ , which are decoded to obtain $\tilde{x}$ . At this point, the missing regions have been filled through multiple steps of denoising, and the denoised output will be as close as possible to the true data distribution, with the observed regions remaining unchanged. This output can directly serve as the imputed result for further analysis.
    Through these two steps—the training phase and the inference phase—the Med-Diffusion model effectively learns to recover the missing parts of samples with missing values, providing an accurate and efficient imputation function *f*.
  f. **Post-processing.** The processed input is then used to train the model. After obtaining the raw output, different processing schemes require different recovery processes.

For one-hot encoding, the index of the largest element is selected as the model’s inferred category. The model outputs a set of continuous values corresponding to the scores or activation values for each category. Categorical Variables: During prediction, the category corresponding to the largest index in the output vector is selected as the final predicted category. Numerical Variables: Since there is no encoding process for numerical variables, the model output is directly taken as the recovered numerical value.

For simulated bit encoding, if an output element is greater than 0, each output element is converted to 1; otherwise, it is converted to −1. The model output consists of continuous values corresponding to the simulated bit encoding. For subsequent categorical variables, each output element is thresholded and set to 1 if it is greater than 0, or −1 otherwise; then, the encoding vector is decoded back to the original category using a predefined mapping. Numerical Variables: Similar to one-hot encoding, the output is directly restored to a continuous value.

In the FT scheme (feature tokenization), we need to recover both numerical and categorical variables from the embeddings [27,28]. The model outputs an embedding vector of the same dimension.

**Categorical Variables:** We calculate the Euclidean distance between the Med-Diffusion output and each categorical embedding. The closest embedding (1-nearest neighbor) is selected as the final model output.

**Numerical Variables:** Since they have not undergone an encoding process, the model’s output is directly used as the recovered numerical value.

**FT Decoding Example for Categorical Variables:** To predict sleep stages (awake, light sleep, and deep sleep), each category has an “embedding vector” (a list of numbers). For example, see the following:

Category 1 embedding vector: $e_{1}=\left(0.2,0.1\right)$ . Category 2 embedding vector: $e_{2}\;=\;\left(0.6,-0.2\right)$ . Category 3 embedding vector: $e_{3}=\left(-0.4,0.7\right)$ . After the CSDI model performs calculations, it outputs a predicted embedding vector $e_{\mathrm{pred}}=\left(0.5,-0.3\right)$ . We calculate the Euclidean distance between $e_{\mathrm{pred}}$ and each category’s embedding vector as follows:

$$\begin{array}{cc} d_{1} & =\sqrt{{\left(0.5-0.2\right)}^{2}+{\left(-0.3-0.1\right)}^{2}}=0.5\\ d_{2} & =\sqrt{{\left(0.5-0.6\right)}^{2}+{\left[-0.3-\left(-0.2\right)\right]}^{2}}=\sqrt{0.02}\\ d_{3} & =\sqrt{{\left(0.5+\left(-0.4\right)\right)}^{2}+{\left(-0.3-0.7\right)}^{2}}=\sqrt{1.81} \end{array}$$

Since $d_{2}$ is the smallest (approximately 0.141), we selected Category 2 as the final prediction.

#### 3.3.2. Evaluation Process

To evaluate the performance of *f*, we were given the test input data $Y_{te}={\left\{y_{i}\right\}}_{i=1}^{n}$ and the true values $X_{te}=\left\{x_{i}^{j}\in R:y_{i}^{j}=\emptyset \right\}$ . We defined ${\hat{y}}_{i}^{j}$ as the *j*-th feature imputed by $f(y_{i})$ . Let $M_{j}=\left\{i:y_{i}^{j}=\emptyset \right\}$ denote the index set of missing values for the *j*-th feature, and let $N_{\mathrm{miss}}^{j}=\left|M_{j}\right|$ be the number of missing values for the *j*-th feature.

To compute the error of *f*, if *j* is a numerical variable, we use the Root Mean Square Error (RMSE); if *j* is a categorical variable, we use the error rate (Err):

(5) $$\mathrm{RMSE}\left(j\right)=\sqrt{\frac{\sum_{i\in M_{j}}{\left({\hat{y}}_{i}^{j}-x_{i}^{j}\right)}^{2}}{N_{\mathrm{miss}}^{j}}},$$

(6) $$\mathrm{Err}\left(j\right)=\frac{1}{N_{\mathrm{miss}}^{j}}\sum_{i\in M_{j}}y_{\left[{\hat{y}}_{i}^{j}\neq x_{i}^{j}\right]},$$

where $y_{\left[{\hat{x}}_{i}^{j}=y_{i}^{j}\right]}$ is an indicator function that returns 1 if the condition holds, otherwise 0. This result is used to optimize the prediction function $f_{\mathrm{sample}}\left(\theta \right)$ .

## 4. Experiments

### 4.1. Datasets

Five datasets were used to evaluate various health indicators and disease predictions. The PhysioNet Challenge 2012 dataset [39] comes from The PhysioNet/Computing in Cardiology Challenge 2012. The Wisconsin Breast Cancer dataset is from the UCI Machine Learning Repository on Kaggle [40]. The COVID-19, diabetes, and heart disease datasets are also from Kaggle. It is important to note that the diabetes and COVID-19 datasets contain only binary classification variables, and all numerical variables in the datasets are preprocessed using min-max normalization.

PhysioNet Challenge 2012 (PhysioNet): PhysioNet is a publicly available medical dataset that includes data from 12,000 ICU hospital admissions. All patients are adults admitted to cardiac, medical, surgical, or trauma ICUs for various reasons. Each record consists of approximately 48 h of multivariate time series data, which captures 37 metrics, such as respiratory rate and blood glucose, from multimodal sensor data during different time periods of the patient’s hospital stay. Each observation is associated with a timestamp in hours and minutes, indicating the observation time since the patient was admitted to the ICU. For example, a timestamp of 35:19 means the observation was made 35 h and 19 min after the patient’s ICU admission.

**Indicators of Heart Disease** [41]: This dataset integrates data from 401,958 adult respondents in the 2020 BRFSS survey (Kaggle, 2021), including heart disease diagnoses (yes/no) and 14 key predictors (e.g., smoking history, BMI, and exercise frequency). Of the samples, 8.1% were diagnosed with heart disease. The data has been weighted by the CDC to reflect the U.S. population distribution. The focus of this study was on the subgroup aged ≥ 50 years (*n* = 187,342) to match the target population. Researchers can predict heart disease risk and provide early interventions. This dataset is widely used in cardiovascular disease research.

**Diabetes Health Indicators Dataset** [42]: Sourced from the CDC’s BRFSS annual telephone survey (Kaggle, 2021), this dataset includes health indicators from 253,680 respondents. The data covers diabetes diagnosis (Type 1/2/gestational/no), BMI, blood pressure, cholesterol levels, and other clinical and behavioral features. A total of 13.4% of the samples were diabetic. This dataset is extensively used in diabetes prediction research, analyzing health conditions of diabetic patients through these indicators.

**Breast Cancer Wisconsin Dataset** [43]: Provided by the UCI Machine Learning Repository this dataset consists of 569 breast tumor biopsy samples with digital features. Each sample contains 30 cell morphology parameters (e.g., radius, texture, and number of concave points) obtained by pathologists through Fine Needle Aspiration (FNA) image analysis. The dataset is labeled as malignant (212 samples) or benign (357 samples), and it was split into a training and test set with a 7:3 ratio. This dataset is widely used in cancer diagnosis model research, particularly in the fields of machine learning and data mining.

**COVID-19 patient pre-condition dataset**: This dataset was collected by Tanmoy et al. and includes clinical records of COVID-19 patients from medical institutions across multiple countries (Kaggle, 2022). The data covers key indicators, such as underlying conditions (e.g., diabetes and cardiovascular disease), symptom severity, hospitalization methods, and final outcomes (recovery/death), with a sample size exceeding 100,000 cases. We extracted 20 features including age, underlying conditions, and blood oxygen saturation for this study. All data were anonymized by medical institutions and cross-center standardized by a professional team to ensure data authenticity and reliability. This dataset is used for predicting patient prognosis and has provided foundational data support for various studies on patient recovery.

### 4.2. Experimental Details

In our experiment, we used the Python 3.10.9 programming language in conjunction with the PyTorch 2.5.1+cu121 machine learning library to build an efficient diffusion probabilistic model aimed at optimizing the data encoding and generation process. The experimental environment was equipped with an NVIDIA GeForce RTX 2080 Ti graphics card, which significantly accelerates the training of deep learning models through its powerful floating-point computation capabilities.

For the training process of the tabular mixed data imputation task, the optimizer for the diffusion model employs a learning rate of 1× for updating the entire model parameters to ensure training stability. The batch size is set to 64 to balance computational efficiency and sample diversity, avoiding overfitting. The hyperparameter T for the diffusion process is set to 1000, enabling a more detailed simulation of the data diffusion process. A quadratic (quad) schedule is adopted for the diffusion noise range $\beta \in [10^{-4},0.5]$ . The number of training epochs was set to 250 to ensure stable convergence and good generalization with the current data size and model capacity. In terms of model architecture, the diffusion network consists of four layers, each with 128 channels, and it features four heads of multi-head attention. The time embedding dimension was set to 128 and the feature embedding dimension was set to 8. Categorical features were tokenized and jointly modeled with numerical features for training and evaluation. The 500 training cycles ensure that the model sufficiently learns the data characteristics and achieves strong generalization, leading to more accurate predictions in real-world applications. During both the training and evaluation stages, the missing rate was set to 0.2 (test_missing_ratio = 0.2), and five-fold cross-validation was employed for model evaluation.

These experimental settings and parameter configurations ensure that the diffusion probabilistic model demonstrates efficient and accurate performance in missing data imputation tasks. Not only do these settings enhance training efficiency, but they also increase the model’s robustness and reliability in practical applications.

## 5. Results and Discussion

This section presents a performance comparison of Med-Diffusion with several existing methods, including mean/mode imputation, Multiple Imputation by Chained Equations (MICE, linear), Random Forest-based multiple imputation (MissForest), and Generative Adversarial Imputation Networks (GAINs). The performance comparison is based on two evaluation metrics: the Root Mean Square Error (RMSE) and error rate. The RMSE is used to assess the imputation accuracy of numerical variables, while the error rate is used to evaluate the imputation accuracy of categorical variables. Each main value in the table is followed by a bracket containing a decimal number. This number represents the standard error (SE), which serves as an indicator of the reliability of statistical estimates (such as mean and RMSE). A smaller SE indicates more stable estimates with higher confidence.

Table 1 presents a comparison of the RMSE and error rate performance on three mixed-variable datasets (diabetes, COVID-19, and cardiovascular datasets). Med-Diffusion achieved the lowest RMSE on the COVID-19 and cardiovascular datasets, while MissForest achieved the lowest error rate on the diabetes and cardiovascular datasets. For categorical variables, Med-Diffusion uses three different encoding techniques: one-hot encoding, simulated bit encoding, and feature tokenization (FT). The experimental results show that Med-Diffusion with FT achieved the lowest error rate on the COVID-19 dataset, indicating that FT is more effective than the other two techniques in handling categorical variables.

**Figure 4:**
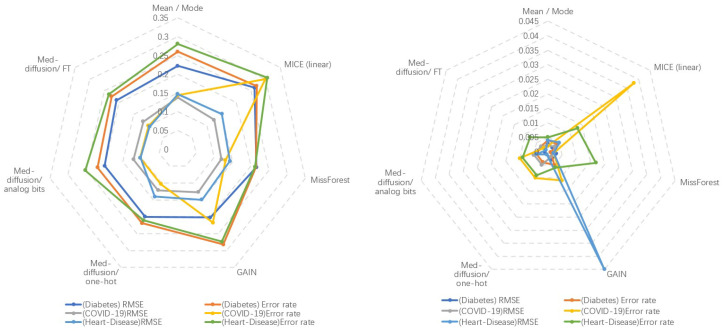
**Left**: Method-wise performance across three datasets. **Right**: Standard error across five baseline and three ablation studies on all datasets.

**Figure 5:**
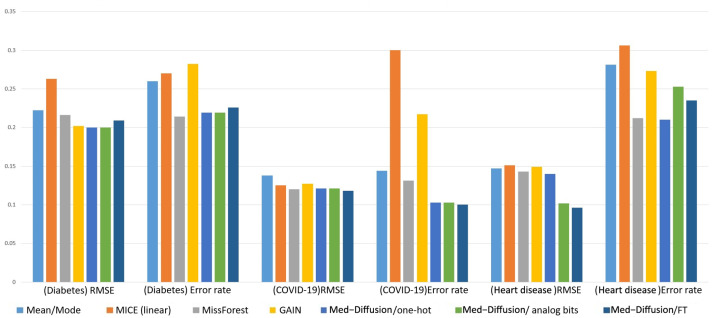
Comparison of the RMSE and error rate performance of the different methods on different datasets.

Table 2 shows a comparison of the RMSE performance on a pure numerical dataset (breast cancer dataset). Med-Diffusion demonstrated the best performance on this dataset, highlighting its effectiveness in imputing numerical variables. The comparison results are illustrated in Figure 4 and Figure 5.

### Discussion

This study presents Med-Diffusion, a diffusion-based framework that combines one-hot encoding, simulated bit encoding, and feature tokenization for multimodal medical data imputation, addressing both categorical and numerical variables. To effectively handle categorical and numerical variables, the method explores the integration of these three techniques with the diffusion-based framework. Experimental results show that the feature tokenization (FT) technique performs better in handling categorical variables. This method leverages the latent structure and distribution information of the data, enabling effective imputation even in the presence of missing values. This is particularly valuable for complex, incomplete, and multimodal medical datasets that involve diverse variables and intricate dependencies. The diffusion probabilistic model better captures latent patterns, improving the quality and accuracy of the imputations.

**Comparison Methods** In this experiment, we compared Med-Diffusion with the following four classical missing data imputation methods.

(1) Mean/Mode Baseline Method:

The mean/mode method is a simple imputation strategy. For numerical features, missing values are replaced with the global mean of that feature from the training set, while for categorical features, the most frequent category (mode) is used for imputation. This method does not require training complex models as it simply computes the mean or mode for each column, making it computationally inexpensive. Despite its simplicity, it provides a reasonable reference level for evaluating the performance of more advanced imputation algorithms. In this study, the mean/mode method showed significantly higher RMSE and error rates on mixed-variable datasets compared to deep and iterative models, highlighting the advantages of the other methods.

(2) MICE (Multiple Imputation by Chained Equations) Linear Regression Version [44]:

MICE is a classic iterative multiple imputation method that treats each feature column as an imputation target. It uses all other columns as predictor variables to train a conditional distribution estimator, and it iteratively generates predicted imputed values for each missing value. In the “linear” version, linear regression is used to predict missing numerical feature values, while logistic regression is employed for categorical features. The process is as follows: (1) initialize all missing values (e.g., using mean/mode); (2) train the corresponding regression or classification model for each column and predict missing values; (3) use the newly imputed values to train the next column’s model; and (4) repeat the process until convergence or the preset iteration limit is reached. MICE captures the linear relationships between features and is suitable for datasets with strong correlations between features. However, its limitations lie in its reliance on strong parametric assumptions based on linear and generalized linear models. For the complex nonlinear relationships (e.g., age and disease risk not being a simple linear relationship), higher-order interaction effects (e.g., a drug’s effectiveness being influenced by both genotype and weight), and non-normal data distributions common in medical data, MICE’s modeling capacity is insufficient and may result in inaccurate imputations.

(3) MissForest Random Forest Imputation [11]:

MissForest is another iterative imputation method, but it uses a non-parametric random forest model as a conditional distribution estimator, addressing the representational deficiencies caused by the linear model assumptions. Its core process is similar to MICE: starting with initial imputed values, a random forest is trained for the current feature to be imputed using other (imputed) features as inputs to predict the missing values and update the imputed values. This process is repeated until the error converges. Since random forests naturally handle mixed-type data (numerical and categorical) and have strong capabilities for modeling nonlinear and interaction relationships, MissForest shows excellent robustness for complex and diverse tabular data. However, its imputation process is autoregressive, meaning features are imputed sequentially, one by one, which may lead to error propagation and accumulation during iterations (errors in early features directly affect subsequent feature models’ training). Moreover, it approximates the joint distribution via the chain rule, rather than directly modeling the joint distribution of the data, which may limit its ability to generate globally consistent and diverse samples.

(4) GAINs (Generative Adversarial Imputation Networks) [12]:

GAINs is a deep generative model based on GANs, where the missing data imputation problem is treated as an adversarial learning task. The framework consists of a generator and a discriminator: the generator receives input with masks (where missing values are randomly set as noise) and attempts to generate imputed values, while the discriminator learns to distinguish real observations from the “pseudo-imputed values” generated by the generator. During training, the generator and discriminator engage in a game where the generator tries to deceive the discriminator, ultimately learning an imputation strategy that matches the true data distribution. Compared to traditional iterative methods, GAINs captures more complex joint distributions and higher-order correlations, providing significant advantages for high-dimensional heterogeneous data. However, the game between the generator and discriminator requires fine hyperparameter tuning. Compared to diffusion models, GANs theoretically have weaker distribution coverage and may fail to fully learn the true distribution of complex multimodal medical data, potentially overlooking some “rare but plausible” imputed values. The performance may not meet expectations when dealing with non-time series data.

(5) CSDI [45]:

CSDI is an innovative method for imputing multivariate time series data. It uses a score-based diffusion model conditioned on the observed data, making it particularly suitable for missing value imputation. Compared to previous score-based methods, CSDI enhances the imputation performance by explicitly training for the imputation task and leveraging the correlations between observations. In CSDI, the model starts from random noise and gradually transforms the noise into reasonable time series values through a reverse diffusion process. The reverse process is conditioned on the observed values, allowing it to better capture the temporal dependencies and feature relationships in the time series. Unlike earlier models that approximate conditional distributions, CSDI directly learns the conditional distribution, providing more accurate imputations. However, CSDI is primarily designed to handle continuous data and does not fully address the importance of categorical variables in data imputation, lacking direct handling capability for multimodal datasets (e.g., categorical variables). Additionally, the score-based diffusion process requires more samples and more complex computations, potentially leading to longer training times.

## 6. Conclusions

Our results demonstrate that Med-Diffusion effectively addresses missing data, significantly enhancing the availability and diversity of multimodal medical datasets—critical for accurate risk assessment and improved surgical outcomes. By incorporating techniques such as one-hot encoding, simulated bit encoding, and feature tokenization, Med-Diffusion enhances the model’s adaptability to both numerical and categorical data types. The framework generates high-quality synthetic data, effectively imputes missing values, and improves the overall data quality, making it a valuable tool for healthcare applications.

## Figures and Tables

**Table 1:** Comparison of the RMSE and error rate performance on three mixed-variable datasets (Diabetes, COVID-19, and Heart Disease indicators). It should be noted that, for datasets without multi-category variables, one-hot encoding and simulated bit encoding are equivalent. The bold numbers represent the state-of-the-art results.

	Diabetes		COVID-19		Heart Disease	
	**RMSE**	**Error Rate**	**RMSE**	**Error Rate**	**RMSE**	**Error Rate**
Mean/Mode	0.222 (0.003)	0.260 (0.004)	0.138 (0.002)	0.144 (0.002)	0.147 (0.004)	0.281 (0.005)
MICE (linear)	0.263 (0.002)	0.270 (0.004)	0.125 (0.003)	0.300 (0.038)	0.151 (0.005)	0.306 (0.013)
MissForest	0.216 (0.003)	**0.214 (0.001)**	0.120 (0.002)	0.131 (0.002)	0.143 (0.002)	**0.212 (0.017)**
GAINs	0.202 (0.003)	0.282 (0.005)	0.127 (0.002)	0.217 (0.011)	0.149 (0.045)	0.273 (0.006)
Med-Diffusion/one-hot	**0.200 (0.001)**	0.219 (0.004)	0.121 (0.005)	0.103 (0.010)	0.140 (0.001)	0.210 (0.009)
Med-Diffusion/simulated bits	**0.200 (0.001)**	0.219 (0.004)	0.121 (0.005)	0.103 (0.010)	0.102 (0.004)	0.253 (0.009)
Med-Diffusion/FT	0.209 (0.002)	0.226 (0.003)	**0.118 (0.003)**	**0.100 (0.002)**	**0.096 (0.001)**	0.235 (0.008)

**Table 2:** Comparison of the RMSE and error rate performance on three mixed-variable datasets (Diabetes, COVID-19, and Heart Disease indicators). It should be noted that, for datasets without multi-category variables, one-hot encoding and simulated bit encoding are equivalent.

	Mean		MICE (Linear)		MissForest		GAINs		CSDI		Med-Diffusion	
	**RMSE**	**MAE**	**RMSE**	**MAE**	**RMSE**	**MAE**	**RMSE**	**MAE**	**RMSE**	**MAE**	**RMSE**	**MAE**
PhysioNet	0.251 (0.013)	0.2002	0.214 (0.013)	0.1707	0.170 (0.012)	0.1356	0.168 (0.010)	0.1340	0.177 (0.027)	0.1412	0.150 (0.008)	0.1197
Breast Cancer	0.263 (0.009)	0.2097	0.154 (0.011)	0.1229	0.163 (0.014)	0.1301	0.165 (0.006)	0.1317	0.195 (0.012)	0.1556	0.143 (0.005)	0.1141
Heart Disease	0.237 (0.012)	0.1891	0.167 (0.016)	0.1332	0.175 (0.010)	0.1396	0.183 (0.008)	0.146	0.169 (0.019)	0.1348	0.153 (0.006)	0.1221

## Data Availability

The original contributions presented in this study are included in the article. Further inquiries can be directed to the corresponding authors.
