# Correction: Cheng et al. Med-Diffusion: Diffusion Model-Based Imputation of Multimodal Sensor Data for Surgical Patients. *Sensors* 2025, *25*, 6175

**DOI:** 10.3390/s26051554

**Published:** 2026-03-02

**Authors:** Zhenyu Cheng, Boyuan Zhang, Yanbo Hu, Yue Du, Tianyong Liu, Zhenxi Zhang, Chang Lu, Shoujun Zhou, Zhuoxu Cui

**Affiliations:** 1 Shenzhen Institute of Advanced Technology, Chinese Academy of Sciences, Shenzhen 518055, China; zy.cheng@siat.ac.cn (Z.C.);; 2 University of Chinese Academy of Sciences, Beijing 100049, China; 3 Department of Mathematics, University of Shanghai for Science and Technology, 334 Campus, Shanghai 200093, China

## Additional Affiliations

In the published publication [1], there was an error regarding the affiliations for Zhenyu Cheng. In addition to the affiliation “Shenzhen Institute of Advanced Technology, Chinese Academy of Sciences, Shenzhen 518055, China”, the updated affiliations should include: “University of Chinese Academy of Sciences, Beijing 100049, China”. The authors state that the scientific conclusions are unaffected. This correction was approved by the Academic Editor. The original publication has been updated accordingly.
